# A Comparative Study for Measuring Serum Ferritin Levels with Three Different Laboratory Methods: Enzyme-Linked Immunosorbent Assay versus Cobas e411 and Cobas Integra 400 Methods

**DOI:** 10.3390/diagnostics12020320

**Published:** 2022-01-27

**Authors:** Lotfi S. Bin Dahman, Khalid M. Sumaily, Essa M. Sabi, Mohammed A. Hassan, Abeer M. Bin Thalab, Asrar S. Sayad, Saleh M. Bin Kolaib, Fatima M. Alhadhrmi

**Affiliations:** 1 Department of Medical Biochemistry, College of Medicine, Hadhramout University, Mukalla 50511, Yemen; 2 Clinical Biochemistry Unit, Pathology Department, College of Medicine, King Saud University, Riyadh 11461, Saudi Arabia; ksumaily@ksu.edu.sa (K.M.S.); esabi@ksu.edu.sa (E.M.S.); 3 Department of Medical Basic Sciences, College of Medicine, Hadhramout University, Mukalla 50511, Yemen; maah3512@gmail.com; 4 Department of Pathology, College of Medicine, Hadhramout University, Mukalla 50511, Yemen; Drabeerm2006@yahoo.com; 5 Department of Obstetrics and Gynecology, College of Medicine, Hadhramout University, Mukalla 50511, Yemen; Dr.asrarsaleh@gmail.com; 6 BSc Candidate of Laboratory Medicine, College of Medicine, Hadhramout University, Mukalla 50511, Yemen; salehkleip@gmail.com (S.M.B.K.); fatimamohsen1997301@gmail.com (F.M.A.)

**Keywords:** ferritin, enzyme-linked immunosorbent assay, immunoturbidimetric, immunochemiluminescence

## Abstract

Different laboratory methods are used to measure serum ferritin levels as a marker of iron status in the general population. This study aimed to compare serum ferritin levels using enzyme-linked immunosorbent assay (ELISA) versus immunochemiluminescence (Cobas e411) and immunoturbidimetric (Cobas Integra 400) methods in terms of sensitivity, specificity and accuracy, and whether they can be used interchangeably. A comparative cross-sectional study enrolled one hundred and six adult Yemeni patients (33 males and 73 females) aged 18–55 years, recruited from the dermatology and cosmetic center of Hadhramout Modern Hospital, Mukalla, Yemen. Serum ferritin levels were measured using ELISA, Cobas e411, and Cobas Integra 400 methods. For method comparison, a paired-sample *t*-test was used. For the consistency between the three methods, they were analyzed with regression and Pearson correlation coefficient. For determining accuracy, a receiver operating curve (ROC) was used. Bias error between the methods was determined through a Bland–Altman plot analysis. Our results did not show any significant statistical difference between ELISA and Cobas e411 (52.55 ± 7.4 µg/L vs. 52.58 ± 7.5 µg/L, *p* = 0.967), while there were significantly higher values from Cobas Integra 400 results than Cobas e411 (56.31 ± 7.8 µg/L vs. 52.58 ± 7.5 µg/L, *p* < 0.001) and ELISA (52.55 ± 7.4 µg/L vs. 56.31 ± 7.8 µg/L, *p* < 0.001). According to the correlation coefficient and linear regression analysis, a strong association between ELISA with Cobas e411 (r = 0.993, *p* < 0.001) and Cobas Integra 400 results (r = 0.994, *p* < 0.001) were revealed. For determining accuracy, Cobas e411 and Cobas Integra 400 results showed higher sensitivity (92.0%; 90.0%) and specificity (97.7%; 99.9%) respectively. Additionally, the Bland–Altman plot analysis showed a high agreement between the ELISA and Cobas e411 methods (bias: −0.035). In contrast, there was a low agreement between the ELISA and Cobas Integra 400 methods (bias: −3.75). Similarly, the agreement between Cobas e411 and Cobas Integra 400 methods was low (bias: −3.72). Serum ferritin levels were measured by Cobas e411, and Cobas Integra 400 methods were strongly correlated with the ELISA results, with higher sensitivity, specificity, and accuracy. However, further investigations with larger samples are required for improved accuracy and more precise results, and to determine whether they can be used interchangeably.

## 1. Introduction

Ferritin is the storage form of iron present mainly in the liver, spleen, and bone marrow, and it is used in iron recycling for hematopoiesis.

As a reflection of iron storage in healthy people, a little amount of iron is found in the plasma and serum of humans [1,2]. Because a low serum ferritin level is commonly used as a marker for iron depletion [2], interpreting serum ferritin values in the presence of acute or chronic inflammation [3,4] could be difficult, as ferritin remains raised in iron-overload conditions and inflammation [5,6,7]. With the further usage of ferritin level being widely employed as a marker of iron stores and status, it is vital to identify if all of the methods for determining ferritin levels can detect and discriminate all conceivable iron states, as well as each method’s comparability among measurement systems.

Serum ferritin concentrations as a marker of iron status are measured using a variety of laboratory procedures. Regarding biological standardization, the World Health Organization (WHO) produced reference materials for developing tests and evaluating laboratory performance. There have been at least three reference materials developed: the first (liver), the second (spleen), and the third (recombinant) [8,9,10]. As a result, the WHO is revising its serum ferritin guidelines to assess iron status [11,12]. Furthermore, the WHO acknowledges that ferritin is commonly measured in serum/plasma using immunoassays; nevertheless, there are no clear recommendations on the variability of analytical procedures [13].

These methods are the enzyme-linked immunosorbent assay (ELISA), immunochemiluminescence, the microparticle enzyme immunoassay (MEIA), and microarray-based technologies [14,15,16].

The enzyme-linked immunosorbent assay (ELISA) is one of the most sensitive immunoassays available for ferritin detection. The typical detection range for se-rum/plasma ferritin by ELISA was around 0.01–0.1 ng, but its sensitivity depends on the particular characteristics of the antibody–antigen interaction. At the same time, some substrates have yielded enhanced chemiluminescent or fluorescent signals to improve ferritin results, where sensitivity and specificity of this technique were 91%, and 83%, respectively [17]. Moreover, immunoturbidimetric and immunochemiluminescence methods were widely used for serum ferritin detection [17].

Since these laboratory methods are used with different test principles, reference ranges, and detection limits, this study therefore aimed to compare serum ferritin levels measured by ELISA, as the reference method, versus the Cobas e411 and Cobas Integra 400 methods, in terms of sensitivity, specificity and accuracy, and whether they can be used interchangeably.

## 2. Materials and Methods

### 2.1. Study Design and Patient Selection

A total of one hundred and six adult Yemeni patients, 33 males and 73 females, were randomly enrolled in a comparative cross-sectional study. The patients were recruited from the dermatology and cosmetic center of Hadhramout Modern Hospital, Mukalla, Yemen, from 1 March to 30 June 2020. Most patients suffered from hair loss problems. Based on the WHO cut-off values of ferritin [13] iron deficiency—deficient (ferritin levels less than 15 µg/L), normal iron (ferritin levels 15–150 µg/L) and iron overload (ferritin levels greater than 150 µg/L)—only 11.4% of patients had iron overload, and the remaining had normal iron (44.3%) and iron deficiency (44.3%). Both cut-off values were determined using ELISA, Cobas e411, and Cobas Integra 400 methods. Patients with comorbidities (chronic inflammation, liver diseases, renal diseases, immunological diseases, malignancies) and prolonged iron supplement intake were excluded. Samples with hemolysis, lipemia, and jaundice were also excluded. After consenting participants, the study was approved by the Ethics Committee of the Medicine College, Hadhramout University, Yemen, according to the Declaration of Helsinki.

### 2.2. Data Collection and Tools

The data was collected using a self-administrated pretested questionnaire collected by the Student of Medical Laboratory Sciences, Department of College of Medicine and Health Sciences, Hadhramout University. The questionnaire focused on sociodemographic (age and sex, iron status, and severity of infection/inflammation), medical history (chronic inflammation, liver diseases, chronic renal diseases, immunological diseases, and malignancies), and the ferritin detection methods used.

### 2.3. Blood Sample Collection and Serum Ferritin Measurement

Each participant had ten milliliters of venous blood drawn and quickly transported to the laboratory. After that, the serum was separated and placed in freezers at −20 °C until the analyses were completed.

Thawing freezing was avoided by dividing them into aliquots. Serum ferritin levels were measured using STAT FAX 4700 ELISA (Awareness Technology, Inc., Palm City, FL, USA), Cobas e411, and Cobas Integra 400 methods (Roche Diagnostics, CH-6343 Rotkreuz, Switzerland). The investigations were performed in the Hadhramout Modern Hospital Laboratories, Mukalla, Yemen.

### 2.4. Enzyme-Linked Immunosorbent Assay (ELISA) Method

We used a solid-phase sandwich assay based on a streptavidin-biotin principle described by the manufacturer’s constructions. In brief, 96 wells were coated with 200 μL mouse anti-ferritin antibody (Cat. No.: RCD012R, BioVendor Laboratory Medicine Inc., Karasak, Czech Republic). Then, 20 µL of each calibrator, control, and serum was added into the corresponding labeled wells in duplicate, and 200 µL of the conjugate working solution (mouse anti-ferritin monoclonal antibody-horseradish peroxidase conjugate) was added into wells and incubated on a shaker (200 rpm) for 30 min at room temperature. After incubation, the wells were washed five times with 300 µL of washing solution (phosphate-buffered saline). After washing, 150 µL of TMB substrate (tetramethylbenzidine) was added to each well and incubated for 20 min at room temperature. After incubation, 50 µL/well of stop solution (1 M H_2_SO_4_) was added. The density of the color produced was measured at 45 nm using a microplate reader. A standard curve was generated, and values for unknown samples were extrapolated.

### 2.5. Cobas e411 Method

First incubation (9 min): a sandwich complex was formed by mixing 10 μL of a sample with a monoclonal antibody specific to ferritin marked with biotin, and a monoclonal antibody specific to ferritin marked with ruthenium. After adding streptavidin-covered microparticles, the complex was coupled to a solid phase by biotin and streptavidin interaction. The reaction mixture was aspired into a measurement cell, where microparticles were magnetically caught by the electrode surface. A photomultiplier was used to monitor chemiluminescence emission caused by voltage application on electrodes.

### 2.6. Cobas Integra 400 Method

Human-driven ferritin showed agglutination with latex particles covered with anti-ferritin antibodies in the expanded particle surface immunoturbidimetric test. Precipitation was turbidimetric at 542 nm. General characteristics of the methods used are summarized in Table 1.

### 2.7. Statistical Analysis

For the statistical analysis, SPSS version 21 was used. Serum ferritin level is presented as mean and standard deviations. Number and percentage were used for categorical variables. The Shapiro test analyzed the normal distribution of the variables. A paired sample *t*-test was performed to show the mean difference of the methods. The relationship between the methods was assessed using correlation coefficient and linear regression analysis. Receiver operating curve (ROC) analysis was used to determine sensitivity, specificity, and accuracy. Bland–Altman plot analysis [18] was used to estimate the bias error between the approaches. The difference in means between the two measures of ferritin level (g/L) was defined as bias and presented as mean SD, and 1.96 SD was stated as the bounds of agreement (95 percent confidence intervals of the bias). If the bias between two approaches was minimal and the limits of agreement were narrow, the two methods were interpreted as equal [18]. These methods follow the measurement procedure recommendations for method comparison studies published by the Clinical and Laboratory Standards Institute in 2013 [19]. We conducted the statistical analysis at 95% confidence level, and *p*-value < 0.05 was considered statistically significant.

## 3. Results

### 3.1. Baseline Characteristics of the Study Participants

The baseline characteristics of the study participants are presented in Table 1. Overall, there were 106 participants—33 were male (31.1%), and 73 were female (68.9%)—with a mean age of 31.57 ± 9.1 (Table 2). Most participants had iron deficiency (44.3%) or normal iron (44.3%). The remaining had an iron overload (11.4%).

### 3.2. Comparison of the Mean Serum Ferritin Concentrations between the Methods

Our study did not show any significant statistical difference between the ELISA and Cobas e411 results (52.55 ± 7.4 µg/L vs. 52.58 ± 7.5 µg/L, *p* = 0.967). However, serum ferritin levels were significantly higher from Cobas Integra 400 results than Cobas e411 and ELISA (56.31 ± 7.8 µg/L vs. 52.58 ± 7.5 µg/L, *p* < 0.001; 52.55 ± 7.4 µg/L vs. 56.31 ± 7.8 µg/L, *p* < 0.001, respectively). Table 3 indicates an acceptable precision between the ELISA and Cobas e411 results.

### 3.3. Sensitivity, Specificity, and Accuracy of Methods for Serum Ferritin Measurement

Furthermore, the ROC curve indicates the overall sensitivity and specificity of ELISA and Cobas Integra 400 Plus, compared with Cobas e411 as the reference method, using the ROC analysis at a 95% confidence interval (Table 4). Although the sample was small, the Cobas e411 showed a significant agreement with the ELISA (89.4%) and Cobas Integra 400 methods (89.4%). For determining accuracy, the sensitivity of the Cobas e411 method was 92.0%, higher than the Cobas Integra 400 method (90.0%). Our study revealed that the Cobas Integra 400 results had the highest specificity (99.9%).

### 3.4. Association between Methods Using Corelation Coefficient and Linear Regression Analysis

**Figure 1:**
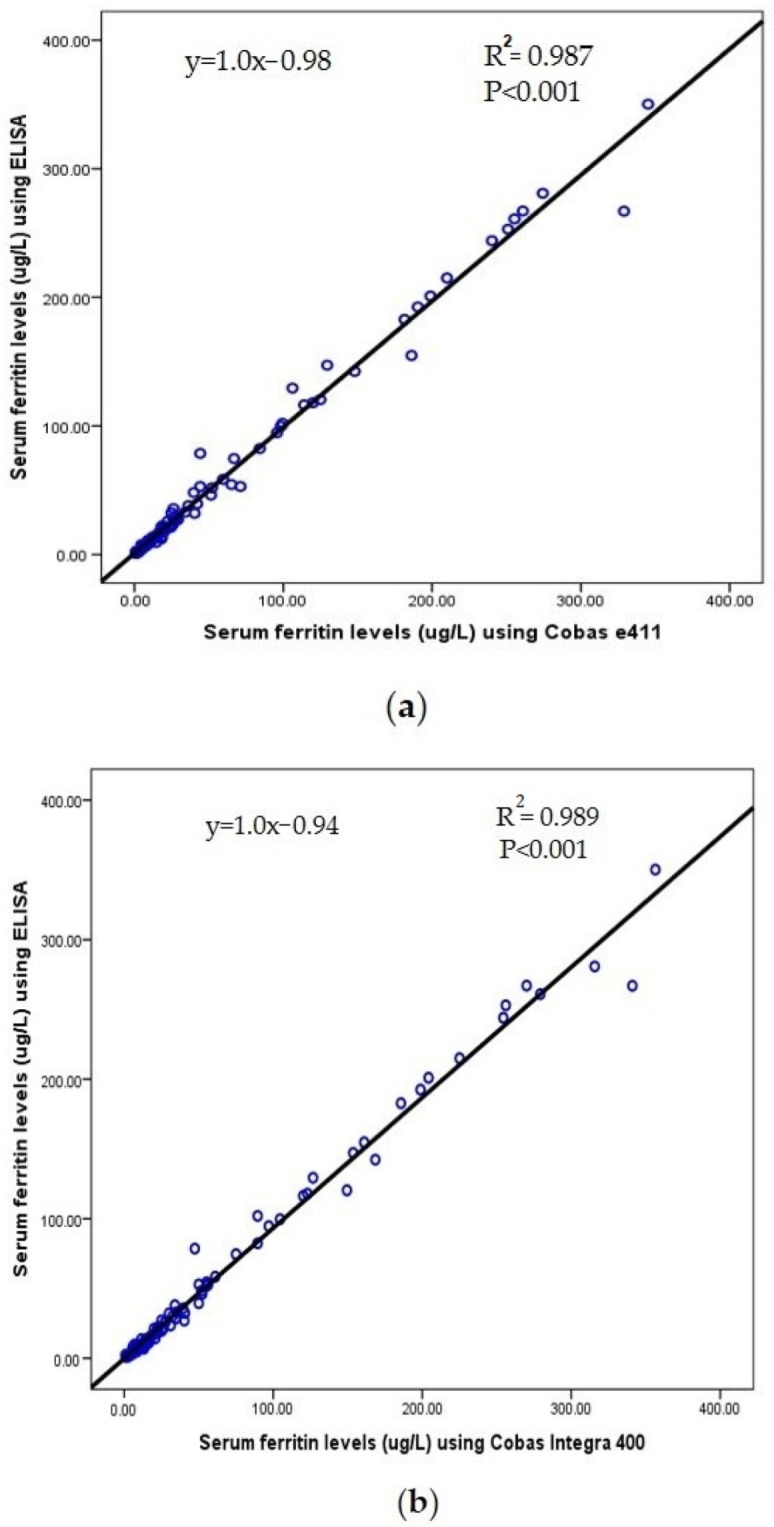

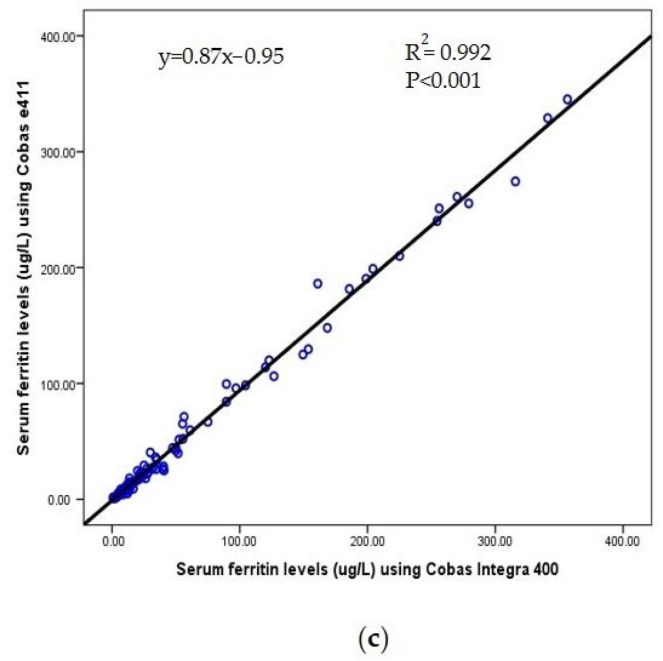
(**a**) Association between ELISA and Cobas e411 methods using linear regression analysis; (**b**) association between ELISA and Cobas Integra 400 methods using linear regression analysis; (**c**) association between Cobas e411 and Cobas e411 methods using linear regression analysis.

According to the results of correlation coefficient, there was a positive correlation between ELISA as the reference method with Cobas e411 (r = 0.993, *p* < 0.001) and Cobas Integra 400 methods (r = 0.994, *p* < 0.001) (Figure 1a,b). Moreover, we found a positive correlation between the Cobas e411 and Cobas Integra 400 results (r = 0.996, *p* < 0.001) (Figure 1c). In contrast, the linear regression equation for serum ferritin levels measured with ELISA compared to the Cobas e411 and Cobas Integra 400 methods (y = 1.0x − 0.98; R^2^ = 0987, y = 1.0x − 0.94; R^2^ = 0.989, respectively), while the results between the Cobas e411 and Cobas Integra 400 methods were: y = 0.87x − 0.95; R^2^ = 0.992.

### 3.5. Agreement between the Methods Using Bland–Altman Plot Analysis

**Figure 2:**
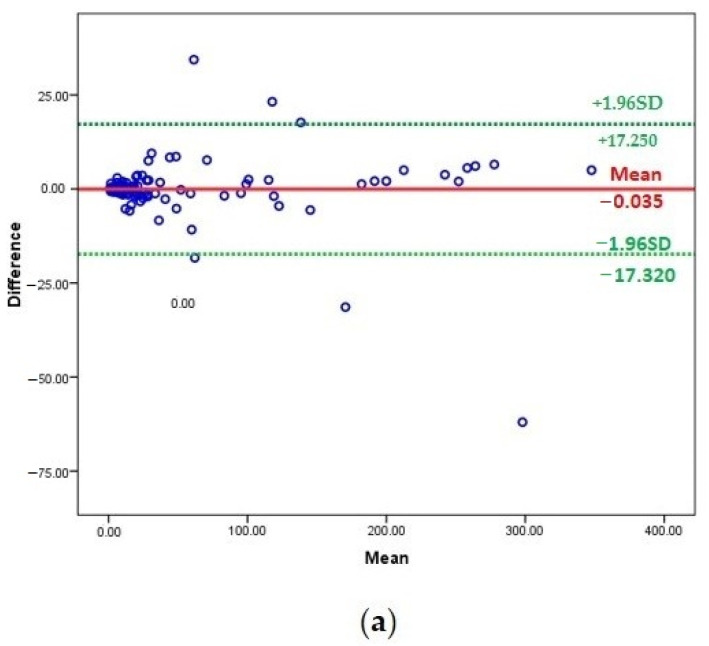

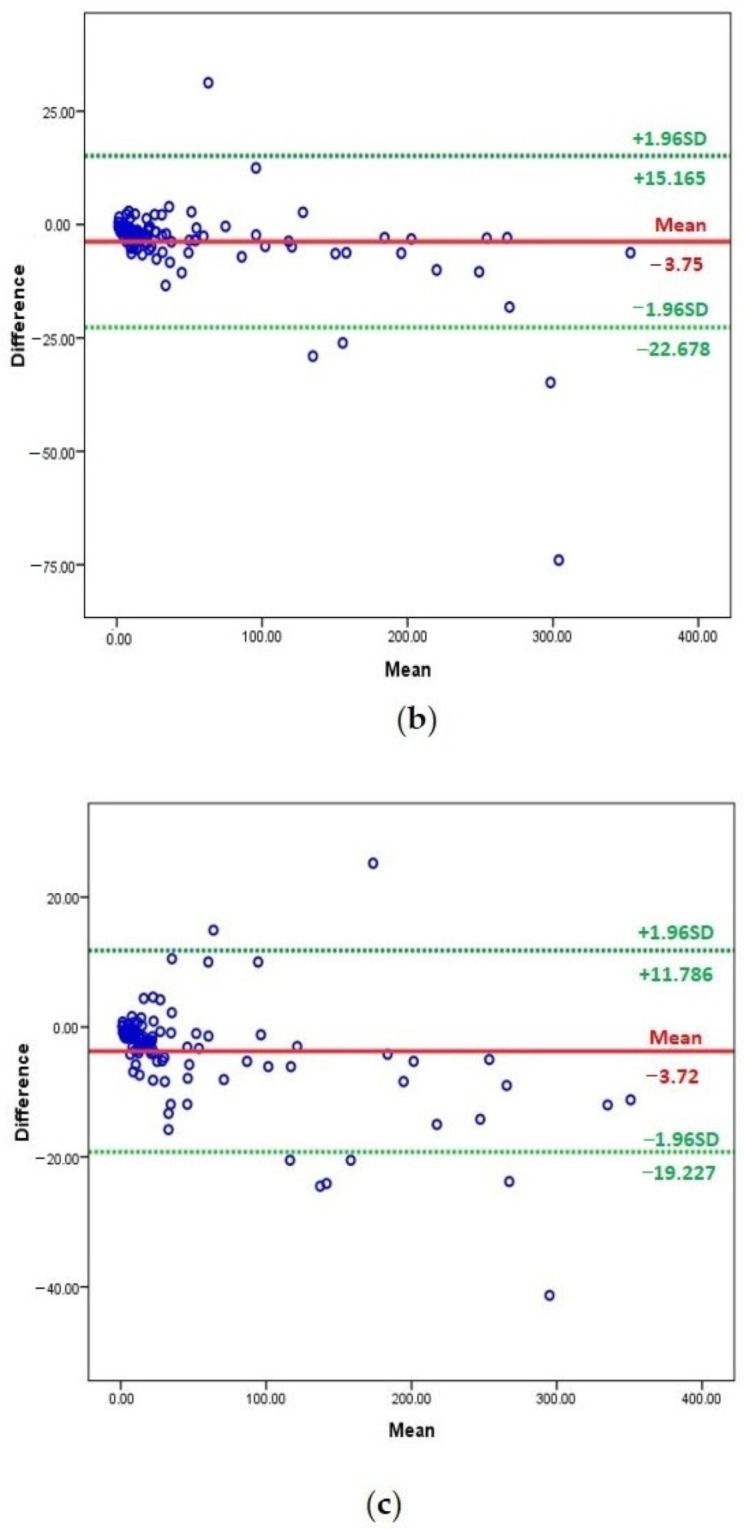
(**a**) Association between ELISA and Cobas e411 methods using Bland–Altman plot analysis; (**b**) association between ELISA and Cobas Integra 400 methods using Bland–Altman plot analysis; (**c**) association between Cobas e411 and Cobas e411 methods using Bland–Altman plot analysis.

For agreement between the compared methods, the Bland–Altman plot analysis showed high agreement between the ELISA and Cobas e411 methods (bias: −0.035) (Table 5 and Figure 2a). In contrast, there was a low agreement between the ELISA and Cobas Integra 400 methods (bias: −3.75). Similarly, the agreement between Cobas e411 and Cobas Integra 400 methods was low (bias: −3.72) (Table 5 and Figure 2b,c).

## 4. Discussion

Measuring ferritin in human serum or plasma in the general population is essential to investigate the prevalence and distribution of iron deficiency and iron overload, thus leading to proper intervention and therapy, and the ability to evaluate the impact and safety implemented in public health programs. The comparability of ferritin results from patient to patient for the differential diagnosis of iron deficiency overload has a critical role in clinical decisions and the appropriate use of resources. Furthermore, comparing data from various surveys performed by different methods should be possible.

Different laboratory methods were developed to quantify the ferritin levels in human serum or plasma from the above findings. The majority were based on antigen–antibody reactions. Moreover, immunoturbidimetric and immunochemiluminescence methods were developed. Recently, chemical autoanalyzer devices for the determination of human ferritin in serum/plasma have been created. The detection method for these devices varies, but is mainly based on turbidimetry and the chemiluminescence method. From the above findings, the present study used three different laboratory methods for serum ferritin level determination: antigen–antibody reaction (ELISA); immunoturbidimetric (Cobas Integra 400); and immunochemiluminescence (Cobas e411).

It is essential to highlight that using the correlation, intercept, slope, and agreement indicators in the statistical analysis indicates the relationship or the linearity between the compared methods and the agreement between these methods. The first result of the present study, regarding whether there was a significant difference between methods for measuring serum ferritin concentrations, revealed that the difference was statistically significant between ELISA with Cobas Integra 400 results. However, no significant difference was shown between the ELISA and Cobas e411 results. Secondly, the results of the methods were consistent according to the correlation coefficient and linear regression analysis. Similarly, human serum ferritin levels were evaluated with different laboratory methods in previous studies.

In a study by Ince et al. [20], serum ferritin levels were estimated using an unintegrated AU5800 analyzer and a Cobas e601 autoanalyzer. It was found that there was a positive correlation between the serum ferritin-assessed results using different methods, indicating that these methods could be used interchangeably, as the difference between them was within clinically acceptable limits. These findings were in agreement with our study.

For agreement between the methods, our study used the Bland–Altman plot analysis. A significantly different bias was observed between Cobas Integra 400 with ELISA and Cobas e411 results, but no considerable results were observed between ELISA and Cobas e411 in our results. However, there are conflicting results for the agreement between these methods. One study by Dupuy et al. [21] compared both turbidimetric and chemiluminescence methods with the radioimmunoassay (RIA). Their Bland–Altman Plot analysis revealed that the methods employed to compare serum ferritin levels were consistent with one another, indicating that these approaches might be used instead of the RIA method [21]. A recent study by Karakochuk et al. [22] measured serum ferritin concentrations using four different laboratory methods in non-pregnant Cambodian women with iron deficiency. They found that the serum ferritin results were different, using different calibrators, ferritin isoforms, and antibodies, and there was a poor agreement between the ELISA and immunochemiluminescence methods (bias: −11.5 to 44 μg/L) [22]. Furthermore, Zhang et al. [23] compared ferritin samples with varying concentrations in Architect i2000 (Abbott Laboratories, Chicago, IL, USA) and Cobas e601 (Roche Diagnostics) devices with two different methods, and revealed that the average serum ferritin concentrations made in Cobas e601 was around 60.6 ng/mL, which was found to be higher than the average of Architect 2000 autoanalyzer. As a result, both procedures demonstrated a correlation; nevertheless, they cannot be used interchangeably, and patients’ serum ferritin readings should always be obtained using the same method [23,24]. Previously, two studies [25,26] observed a moderate agreement between the ELISA methods versus the chemiluminescence and radiometric methods (bias: −8.0 to 3.7 μg/L and 8.5 to 12 μg/L, respectively). Between chemiluminescence (bias from 34 to 60 g/L and 12 to 71 g/L), Zhang 2015 [23] and Dipalo 2016 [27] reported moderate to poor agreement, but Molinario 2015 [28] and Gomez 2000 [29] found strong agreement between the agglutination and chemiluminescent methods (bias: −6.0 to −7.9 μg/L; 1.5 to 8 μg/L, respectively).

Human ferritin ELISA kits are designed as simple, convenient, and low-cost ferritin measurements in plasma or serum. They produce results that are consistent with commercial immunoassay systems, including values of less than 30 ng/mL, which is the range of interest for those investigating iron deficiency anemia [13]. Their sensitivity of roughly 0.5 ng/mL is also sufficient to quantify ferritin, even in those with very low levels, and it is equivalent to the Abbot Architect immunoassay (1.0 ng/mL) [6]. Thus, these can be performed with minimal equipment. In contrast, the nature of the antibodies used for ferritin measurement in human serum/plasma is essential to recognize the variations in ferritin results.

The majority of ELISA antibodies are polyclonal and produced against full-length native human liver ferritin.

Ferritin is a 24-subunit protein made up of two types of subunits (H and L), and the ratio of the two subunits varies depending on the ferritin isoform [2]. For example, ferritin from the heart contains mostly the H subunit, whereas ferritin from the liver (and also ferritin in plasma) contains mostly the L subunit. As a result, it has been suggested that variations in how the isoform is measured is one possible explanation for variations in ferritin determination results between different assay methods [22], and that information about the ferritin source against which the antibodies used in the assays were generated is essential for comparing results measured using different methods.

## 5. Conclusions

The differences in serum ferritin levels likely reflect different ferritin isoforms, antibodies, and calibrators used across assays by different laboratories. However, serum ferritin concentrations measured by Cobas e411 and Cobas Integra 400 methods are strongly correlated with ELISA results, with a higher sensitivity, specificity, and accuracy. In conclusion, further investigations with larger samples are required for better accuracy, more precise results, and to determine whether they can be used interchangeably.

## Figures and Tables

**Table 1:** General characteristics of the methods.

	ELISA	Cobas e411	Cobas Integra 400
Testing time	60 min	18 min	9 min
Test principle	Sandwich assay	Sandwich assay	FerroZine assay
Calibration	Endpoint	2 point	Endpoint
Sample volume	20 μL	10 μL	8.5 μL
Detection limit	0.44 µg/L	0.5 µg/L	5.0 µg/L
Reference range	Males: 20–250 µg/L Females: 10–120 ug//L	Males: 30–400 μg/L Females: 13–150 μg/L	Males: 59–158 µg/L Females: 37–145 µg/L
Measuring range	0.8–2000 µg/L	0.5–2000 µg/L	5–1000 µg/L
Linear regression	y = 1.03x − 20.12; R^2^ = 0.970	y = 1.0x − 098; R^2^ = 0.987	y = 1.041x − 0.56; R^2^ = 0.999
Limitations	Icterus: significant interference Hemolysis: significant interference Lipemia: no significant interference	Icterus: no significant interference (bilirubin < 1112 mmol/L). Hemolysis: No significant interference (Hb < 0.31 mmol/L). Lipemia: no significant interference.	Icterus: No significant interference. Hemolysis: No significant interference up to Hb 200 mg/dL. Lipemia: no significant interference.

Data on file at the Roche Diagnostics Switzerland (kit) for Cobas e411 and Cobas Integra 400, and BioVendor Laboratory Medicine (equipment) for ELISA. ELISA; enzyme-linked immunosorbent assay.

**Table 2:** Baseline characteristics of the participants.

Participants No. (106)	Mean ± SD	Frequency (*n*)	Percentage (%)
**Age (years):**	31.57 ± 9.1		
**Age categories (years):**			
18–26		37	34.9
27–35		37	34.9
≥36		32	30.2
**Sex:**			
Male		33	31.1
Female		73	68.9
**Iron status:**			
Iron deficiency		47	44.3
Normal		47	44.3
Iron overload		12	11.2

**Table 3:** Comparison of the mean serum ferritin concentrations between the methods.

Participants No. (106).	Mean ± SE	T	*p*-Value
ELISA vs. Cobas e411	52.55 ± 7.4 vs. 52.58 ± 7.5	0.042	0.967
ELISA vs. Cobas Integra 400	52.55 ± 7.4 vs. 56.31 ± 7.8	4.01	<0.001
Cobas Integra 400 vs. Cobas e411	56.31 ± 7.8 vs. 52.58 ± 7.5	4.84	<0.001

Data are presented by mean ± standard error (SE). A paired sample *t*-test was performed to show the mean difference of the methods. ELISA; enzyme-linked immunosorbent assay.

**Table 4:** Sensitivity, specificity, and accuracy of methods for serum ferritin measurement.

Participants No. (106)	Sensitivity (%)	Specificity (%)	(AUC) Accuracy (%)	*p*-Value
Cobas e411	92.0	97.7	98.9	<0.001
Cobas Integra 400	90.0	99.9	99.6	<0.001

ROC indicates the overall sensitivity and specificity of Cobas e411 and Cobas Integra 400 compared to ELISA as the reference method using ROC analysis at a 95% confidence interval. *p* < 0.05 is considered statistically significant. AUC; area under curve, ELISA; enzyme-linked immunosorbent assay.

**Table 5:** Bias, limits of agreement, and correlation coefficients of methods.

Participants (106)	Bias (Mean ± SD)	Limits of Agreement ± 1.96 SD	Correlation Coefficient (r)	Convidence Interval (95% CI)		*p*-Value
				Lower	Upper	
ELISA vs. Cobas e411	−0.035 ± 8.86	−17.32, 17.24	0.993	−1.74	1.67	0.238
ELISA vs. Cobas Integra 400	−3.75 ± 9.65	−22.67, 15.16	0.994	−5.61	1.89	<0.001
Cobas e411 vs. Cobas Integra 400	−3.72 ± 7.91	−19.22, 11.78	0.996	−5.24	2.19	<0.001

ELISA; enzyme-linked immunosorbent assay.

## Data Availability

The research data from the study may be available from the corresponding author on reasonable request.
